# Microbial Platform for Terpenoid Production: *Escherichia coli* and Yeast

**DOI:** 10.3389/fmicb.2018.02460

**Published:** 2018-10-12

**Authors:** Chonglong Wang, Mudanguli Liwei, Ji-Bin Park, Seong-Hee Jeong, Gongyuan Wei, Yujun Wang, Seon-Won Kim

**Affiliations:** ^1^School of Biology and Basic Medical Sciences, Soochow University, Suzhou, China; ^2^Division of Applied Life Science (BK21 Plus), PMBBRC, Gyeongsang National University, Jinju, South Korea; ^3^Department of Marine Science, Qinzhou University, Qinzhou, China

**Keywords:** terpenoid, *Escherichia coli*, yeast, synthetic biology, MEP pathway, MVA pathway, strain engineering

## Abstract

Terpenoids, also called isoprenoids, are a large and highly diverse family of natural products with important medical and industrial properties. However, a limited production of terpenoids from natural resources constrains their use of either bulk commodity products or high valuable products. Microbial production of terpenoids from *Escherichia coli* and yeasts provides a promising alternative owing to available genetic tools in pathway engineering and genome editing, and a comprehensive understanding of their metabolisms. This review summarizes recent progresses in engineering of industrial model strains, *E. coli* and yeasts, for terpenoids production. With advances of synthetic biology and systems biology, both strains are expected to present the great potential as a platform of terpenoid synthesis.

## Introduction

Terpenoids comprise a vast family of the most abundant (>55, 000 known members) natural products with diverse biological functions including cell integrity, hormones, electron transport, and photosynthetic machinery. The extracted products from some herbs or animal liver are realized as flavors, fragrances, colorants, commodity chemicals, vitamins, and pharmaceuticals from ancient time. Nevertheless, their low yields from natural sources limit mass production of terpenoid for industrial application. For instance, *Artemisia annua* (Qinghao) just yields artemisinin of <0.8% by dry biomass weight (Zyad et al., 2018), which severely restricts commercialization of this antimalarial drug. Humans have employed microbes to produce beverages (e.g., *Saccharomyces cerevisiae*) before civilization, and antibiotics (e.g., *Penicillium chrysogenum*) at an industrial scale since the Second World War (Nielsen and Keasling, 2016). Nowadays, advances in metabolic engineering enable us to build microbial cell factories to generate many interesting products, of which *Escherichia coli* and yeast are the well-characterized hosts for efficient and large-scale production. Since Amyris Inc., announced a record low manufacturing cost of $1.75 per liter for farnesene from yeast in 2015, microbial farnesene has attracted a lot of interests from industry^1^. This review describes progress in biosynthesis of the diverse terpenoids in *E. coli* and yeast, where fantastic technologies are harnessed for development of microbial cell factories. It illuminated the great potential of *E. coli* and yeast on tackling a complexity of biosynthesis of the diverse terpenoids.

## Biosynthesis Pathways of Diverse Terpenoids

**FIGURE 1:**
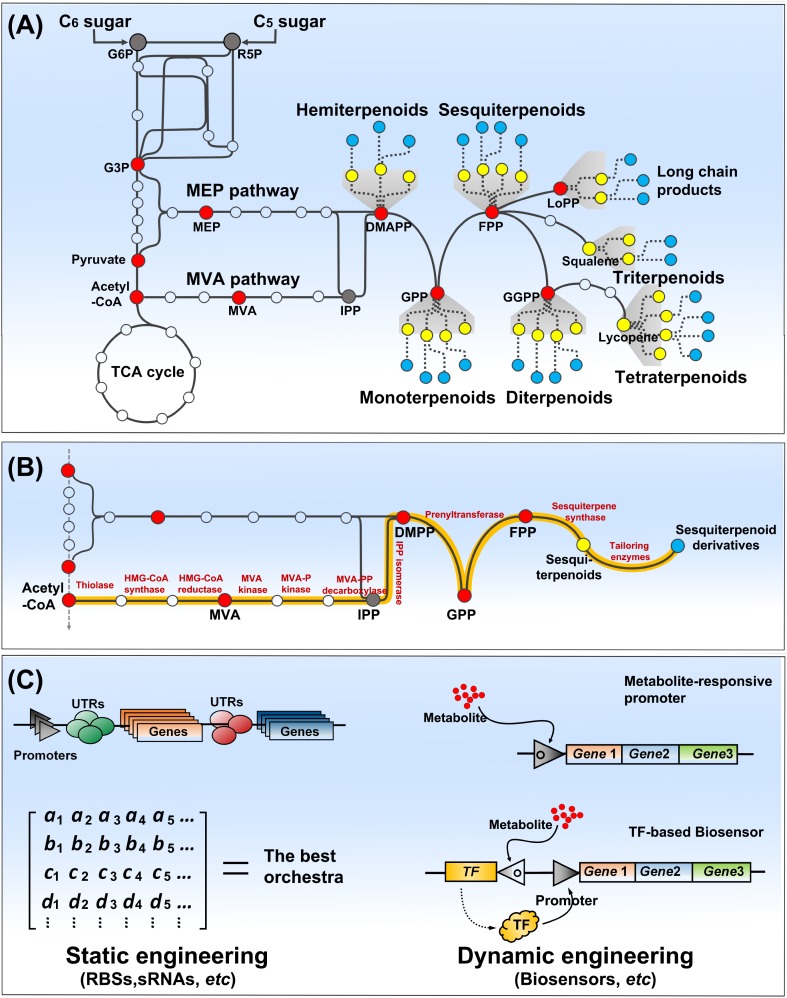
Overview of terpenoids biosynthesis pathway **(A)**, and pathway engineering strategies **(B,C)**. Terpenoids biosynthesis is comprised of carbon assimilation, isoprene unit synthesis, terpenoids backbone synthesis, and terpenoids decoration. C_6_ or C_5_ sugar enters the central pathway of glycolysis. MEP and MVA pathways use central metabolites to initiate synthesis of IPP and DMAPP, the building blocks of terpenoids. Terpenoid synthesis pathways could be assembled and engineered in a tractable host (e.g., *E. coli* or yeasts) to create cell factories for their mass production. Two fashions of static and dynamic engineering have been used to optimize the synthesis pathways and coordinate them with host metabolic network. Static engineering approach generally constructs a matrix of genes, promoters, and regulatory elements (e.g., UTRs) to screen the best orchestra, while dynamic engineering approach relies on biosensors (metabolite response or transcription factor-based) to dynamically control the synthesis pathway. The red dots present the key intermediates of terpenoid biosynthesis, and the yellow and cyan dots present primary terpenenoids and decorated terpenoids, respectively **(A,B)**. TF presents transcription factor **(C)**.

Albeit structurally diverse, skeletons of terpenoids are composed of C_5_ isoprene units which are successively assembled by biogenic isoprene rule. Precursors of the C_5_ isoprene units are isopentenyl diphosphate (IPP) and dimethylallyl diphosphate (DMAPP), which are synthesized by either mevalonate (MVA) pathway in eukaryotes, archaea and a few bacteria or 2-C-methyl-D-erythritol 4-phosphate (MEP) pathway in prokaryotes and plant plastids (Liao et al., 2016; Frank and Groll, 2017). MEP pathway starts from condensation of two glycolytic intermediates, pyruvate and glyceraldehyde-3-phosphate, and MVA pathway from condensation of acetyl-CoAs (**Figure 1A**). Both pathways require energies (ATP) and reductive powers [NAD(P)H] to proceed multi-enzymatic reactions to produce IPP and DMAPP. Given no consideration of energy balance, MEP pathway consumes 1 molecule of glucose per IPP with a carbon molar yield of 0.83, while the MVA pathway consumes 1.5 molecules of glucose per IPP with the lower yield of 0.56 (Eqs. 1, 2) (Schempp et al., 2018). In this scenario, MEP pathway requires 2 ATP and 2 NAD(P)H, while MVA pathway accompanies generation of 3 NAD(P)H. Thus, microorganisms need engineering to use both pathways to attain the carbon and energy balances, which results in a synergy between both pathways for production of terpenoids (Yang et al., 2016).

(1) $$\mathrm{MEP}:\;1\;\mathrm{Glucose}+2\mathrm{ATP}+2\;\mathrm{NAD(P)H}\to 1\;\mathrm{IPP}+{\mathrm{CO}}_{2}\;(Y_{\mathrm{IPP/Glucose}}=0.83\;\mathrm{C-mol}/\mathrm{C-mol})$$

(2) $$\mathrm{MVA}:\;1.5\;\mathrm{Glucose}\to 1\;\mathrm{IPP}+4\;{\mathrm{CO}}_{2}+3\mathrm{NAD(P)H}\;(Y_{\mathrm{IPP/Glucose}}=0.56\;\mathrm{C-mol}/\mathrm{C-mol})$$

Next, prenyltransferases catalyze chain elongation of isoprenyl diphosphates including geranyl diphosphate (GPP, C_10_), farnesyl diphosphate (FPP, C_15_), geranylgeranyl diphosphate (GGPP, C_20_), and even longer chain isoprenyl diphosphates (LoPP, >C_45_), which determine the primary diversity of terpenoids in the chain lengths. Thus, terpenoids are classified to hemiterpenoids (C_5_), monoterpenoids (C_10_), sesquiterpenoids (C_15_), diterpenoids (C_20_), triterpenoids (C_30_), tetraterpenoids (C_40_), and long chain isoprenyl products (**Figure 1A**).

Terpene synthases convert isoprenyl diphosphates to numerous terpenes via carbocationic reaction cascades and/or hybridization of carbon atoms, which generate a myriad of carbon skeletons containing several stereocenters and present their diversity in the structure (Christianson, 2017). The skeletons are further regio- and stereo-selectively decorated by many tailoring enzymes such as cytochrome P450s (P450s), acyltransferase, and glycosyltransferase, which may act in a combinatory manner and finally render various functions to isoprenoids (e.g., artemisinin, taxol and ginkgolide) (King et al., 2016). Therefore, the microbes, for which a good genetic engineering toolbox is available, can be engineered to synthesize valuable terpenoids by assembling of the precursor synthesis, isoprenyl chain elongation, structural rearrangement and tailoring reactions.

## Microbial Hosts for Terpenoid Production

As most terpenoids have been discovered in plants, an extraction from plants is a major source of commercialized terpenoids. However, the plant extraction is compromised by its low productivity due to a slow growth of plants, a low product content in plants, and a limitation in cultivating some species. Even though there is a biotechnological progress in transgenic plants, it still remains difficult to engineer plants for improved isoprenoids contents. Microbes grow fast and do not heavily rely on land/water resources. Thus, they possess a great potential for sustainable mass production of terpenoids over plants. Microbial hosts in industry are required to meet the criteria: (i) a large metabolic potential supporting efficient synthesis of products of interest with robust and fast cell growth, (ii) a well-understood metabolism and well-developed genetic tools (e.g., expression vectors), and (iii) a great capacity to grow on cheap carbon sources. Terpenoids synthesis relies on MEP or MVA pathway for the generation of building units, IPP and DMAPP. Therefore, two representative microbial hosts *E. coli* and *S. cerevisiae*, which are based on MEP and MVA pathways, respectively, have been used for production of diverse terpenoids.

The terpenoids natively produced in *E. coli* are limited to small amounts (e.g., quinones). The native MEP pathway could be less efficient in IPP and DMAPP supply for terpenoid production. *E. coli* has been engineered to improve IPP and DMAPP synthesis by augmenting bottleneck enzymes of MEP pathway or introducing heterologous MVA pathway. *S. cerevisiae* accumulates a large amount of ergosterol, which presents a potential of its MVA pathway for terpenoid production. *S. cerevisiae* has redox systems that allow cytochrome P450 to modify terpenoids skeleton, whereas *E. coli* has none. *S. cerevisiae* is superior to *E. coli* in synthesis of value-added terpenoids with complicated structures. *Yarrowia lipolytica* is another good yeast for production of terpenoids based on MVA pathway because of its abundant acetyl-CoA, the initial substrate of MVA pathway (Zhu and Jackson, 2015). Besides, carotenogenic *Rhodosporidium toruloides* is developed to produce terpenoids from lignocellulosic hydrolysates in that it exerts ability to utilize multiple carbons and tolerates high osmotic stress (Yaegashi et al., 2017; Sundstrom et al., 2018). As many terpenoids have been isolated from *Streptomyces* species (Koksal et al., 2012; Rinkel et al., 2017), the bacteria could also be a potential host for terpenoids production in the future (Phelan et al., 2015; Khalid et al., 2017).

## Metabolic Engineering of *E. coli* and Yeasts for Terpenoid Production

Microbial production of terpenoids can be addressed by introduction of relevant genes for their synthesis into host strains (**Figure 1B**). Owing to a versatility of *E. coli* and *S. cerevisiae*, many strategies of metabolic engineering have been developed and examined to increase terpenoid production in the both model hosts. The engineering strategies can be classified to “static” and “dynamic” according to a regulatory means of synthetic pathway. Both approaches have been applied to terpenoid synthetic pathways for an enhancement of IPP/DMAPP flux, a minimization of byproducts formation, a toxicity reduction of pathway intermediates, etc., Static engineering employs variation of vectors, promoters, ribosome binding sites (RBSs), and genes of interest, which are assembled in a plethora of biological systems, to optimize a synthesis of target products (Ren et al., 2017; **Figure 1C**). Brewer’s yeast has been engineered by combinatory regulation of pathway genes to biosynthesize aromatic monoterpenes that impart hoppy flavor to beer (Denby et al., 2018). Farnesol is a desirable biofuel molecule derived from FPP by phosphatases. By variation of phosphatases, 526 mg/L of farnesol was produced in an engineered *E. coli* overexpressing a membrane integral phosphatase, PgpB (Wang C. et al., 2016). Further phosphatase mining retrieved a cytosolic phosphatase NudB, whose overexpression led to farnesol of 1.42 g/L along with hemiterpenoids isopentenol (Zada et al., 2018). Optimization of plasmids carrying synthesis pathway of terpenoids brought an enhanced production of monoterpenes, 653 mg/L of 1,8-cineole of and 505 mg/L of linalool, from *E. coli* (Mendez-Perez et al., 2017). Oleaginous *Y. lipolytica* is able to synthesize a massive amount of acetyl-CoA, and lipid droplet is supposed to store lipophilic terpenoids. Tuning of promoter strength by promoters shuffling resulted in β-carotene production of 111.8 mg/L in *Y. lipolytica* (Larroude et al., 2018).

However, the static engineering is too laborious to gain a desired performance from an engineered strain due to intermediates toxicity or metabolic burdens often occurring from a mass production of terpenoids, whereas the dynamic engineering can address such adverse circumstances. Thus, there is also a great interest to develop tunable or inducible promoters and small regulatory RNAs (Ghodasara and Voigt, 2017; Marschall et al., 2017; Portela et al., 2017; Trassaert et al., 2017), which could benefit to a dynamic control of synthetic pathways of terpenoids. Biosensors have been developed to sense small molecules (metabolites), which are applied to a transcription control of the committed pathway in response to metabolite abundance (Dekker and Polizzi, 2017). They are built to a simple metabolite responsive promoter or a transcription factor (TF)-based binary system (**Figure 1C**), which are incorporated as a metabolic controller for dynamic flux control (Mannan et al., 2017). For example, FPP repressive promoters were developed to down-regulate FPP biosynthesis and FPP activated promoters could be used to up-regulate expression of amorphadiene synthase converting FPP to amorphadiene. These two promoters were combined in *E. coli* to implement the negative and positive feedback loops of the synthesis and conversion of FPP, which is a critical metabolic node in amorphadiene synthesis. Amorphadiene production of 1.2 g/L was obtained from the engineered *E. coli*, which was a two-fold increase in the production through the dynamic control (Dahl et al., 2013). In *S. cerevisiae*, a large amount of FPP is mainly used for synthesis of ergosterol via squalene. Thus, squalene synthase (Erg9) condensing two FPPs to a squalene could be a critical regulation point to divert FPP flux to synthesis of terpenoids of interests. A dynamic control of Erg9 expression using ergosterol-responsive promoter could alter FPP flux to amorphadiene synthesis (Yuan and Ching, 2015). Biosensors have been developed to provide an additional exquisite regulatory means for tuning of synthetic pathway, and improved in both their dynamic responsive range and substrates spectrum (Rogers et al., 2016; Liu et al., 2017). Natural TFs (AraC and Gal4) were evolved to respond to IPP accumulation (Chou and Keasling, 2013). Their application in controlling zeaxanthin biosynthesis pathway resulted in a successful production of the carotenoid (C_40_) at a titer of 722.5 mg/L (Shen et al., 2016). Synthetic biologists have also designed promoters responding to environmental signals such as pH and quorum sensing (QS) molecules (Tsao et al., 2010; Zargar et al., 2015; Rajkumar et al., 2016). It is capable control terpenoid biosynthesis with the environmental signals, not using expensive inducer molecules. A QS-based promoter system was successfully applied for an inducer free production of bisabolene from *E. coli*, where titer of bisabolene was increased to 1.1 g/L through optimization of LuxR and LuxI expressions (Kim et al., 2017).

## Genome-Level Engineering of Terpenoids Production

**FIGURE 2:**
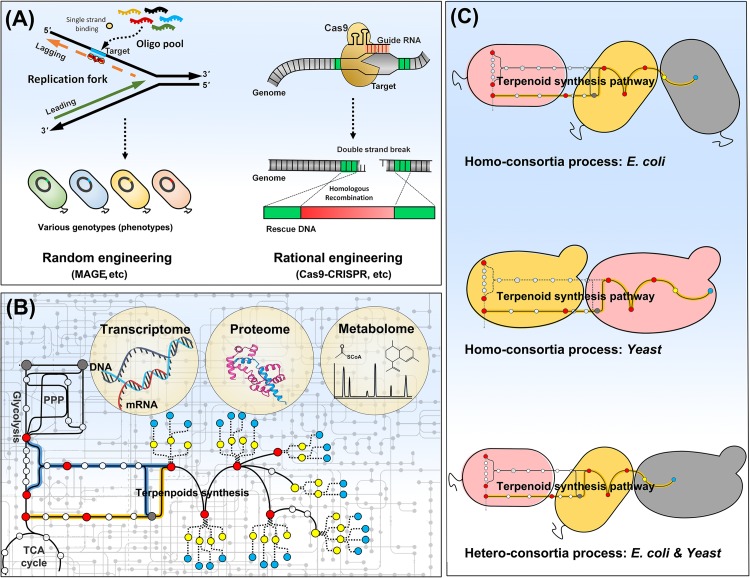
Strain manipulation by genome editing **(A)**, integrated-omics **(B)**, and consortia process **(C)**. Host genome can be evolved by either random (e.g., MAGE) or rational engineering (e.g., Cas9-CRISPR). The integrated-omics approaches can comprehensively elucidate host metabolism, which benefit strain manipulation. The microbial consortia process divides a long complicated pathway into a few short simple pathways, dispersed among microbes in the consortia. It can be built in the same species (homo-consortia) or the different species (hetero-consortia).

Rewiring of metabolic network of host strain is required for a full performance of an engineered pathway for product synthesis, when potential of the engineered production pathway is restricted due to a metabolic characteristics of the host. Many genetic tools are available for genome engineering in *E. coli* and yeasts. Homologous recombination is the most popular method for deletion and replacement of genetic parts. The glyceraldehyde 3-phosphate and pyruvate supply was rebalanced by tuning-down of glyceraldehyde 3-phosphate synthase, which led to a two-fold increase in lycopene production via the MEP pathway in *E. coli* (Jung et al., 2016). The central pathway was rewired in *S. cerevisiae* to synthesize acetyl-CoA with reduced losses of carbon and ATP. As acetyl-CoA was the substrate of the MVA pathway, rewiring of the central carbon metabolism in *S. cerevisiae* resulted in an increase in farnesene production by 25% along with a reduced requirement of oxygen by 75% (Meadows et al., 2016). Genome engineering tools have been developed to simultaneously target multiple genomic loci like as multiplex automated genome engineering (MAGE), and to precisely edit a specific genomic locus without a scar based on the clustered regularly interspaced short palindromic repeats (CRISPR)/Cas9 system (**Figure 2A**). MAGE was applied to optimizing MEP pathway in *E. coli* for lycopene production (Wang et al., 2009). A modified method of MAGE was also successfully applied for β-carotene production in *S. cerevisiae* (Barbieri et al., 2017). As MAGE generates a vast of combinatorial variants in genome, it could be an effective genome engineering tool only when it is combined with a high-throughput screening (colorimetric or fluorescent) system enabling a selection of variants with desired traits. It is also difficult to incorporate large genetic parts (>1 kb) into genome by MAGE. CRISPR/Cas9 is currently powerful and popular genetic tool for precise genome editing (Jiang et al., 2013; Jessop-Fabre et al., 2016). The endonuclease Cas9 combined with guide RNA (gRNA) specifically recognizes a target site in genome, and facilitates site-specific nucleotide base-pair mutations, gene deletions, or large DNA fragment (around 8 kb at least) insertions (**Figure 2A**). β-Carotene synthesis pathway was integrated into *E. coli* genome by CRISPR/Cas9-based method. Both MEP pathway and central metabolic pathways were optimized in the engineered strain, and the final strain was cultured in fed-batch, yielding 2.0 g/L of β-carotene (Li et al., 2015). A set of genomic loci of *Y. lipolytica* have been identified for targeted marker-less integration using CRISPR/Cas9 (Schwartz et al., 2017). An automated platform for multiplex genome-scale engineering in *S. cerevisiae* has been also developed based on CRISPR/Cas9 (Si et al., 2017). Plasmid-based expression of production pathway has a problem of segregational or structural instability of plasmids, which becomes worse in case of inhibitory products to host cells. Moreover, expensive antibiotics is required to exert a selection pressure on host cells for prevention of plasmid loss, which increases a production cost. However, genome-based expression of production pathway could deliver stable and reproducible production with no use of antibiotics, which is important for mass scale production of industry. The top portion of MVA pathway was integrated into genomic loci of *adhE* and *ldhA* in *E. coli*, and *atpFH* genes were deleted to increase glycolytic rate. The genome-engineered strain exhibited both high productivity (∼1.01 g/liter/h) and yield (86.1% of theoretical yield) after 48 h of shaking flask culture (Wang J. et al., 2016). However, engineering of genome integrated production pathway is a cumbersome task compared to plasmid-based engineering. Plasmid is more easy and convenient to modulate genes dosage and to construct various genetic expression cassettes. The genome level engineering for optimization of production pathway was facilitated by CRISPR/Cas9-based convenient chromosomal promoters change, which is successfully applied for bisabolene synthesis in *E. coli* (Alonso-Gutierrez et al., 2018). A heterologous MEP pathway was integrated into genome of *S. cerevisiae* with endogenous MVA pathway. As the MEP pathway does not produce extra NADH which need oxidizing to maintain redox status, the engineered yeast enabled to rely solely on the MEP pathway instead of the MVA pathway for terpenoids biosynthesis under low aeration conditions (Kirby et al., 2016). MVA pathway genes attached with mitochondrial-targeting signal sequences were integrated into genome of *S. cerevisiae* to enable utilization of both cytosolic and mitochondrial acetyl-CoAs via the native cytosolic and the engineered exogenous mitochondrial MVA pathways. The engineered yeast produced 2.5 g/L of isoprene, an increase of 1.6-fold by the mitochondrial MVA pathway (Lv et al., 2016). A comprehensive understanding of whole metabolic networks is a prerequisite to rational editing of genomes or design of biosynthesis pathways (Campbell et al., 2017). Metabolic engineers paid efforts to interpret cellular phenomena at a systems level by measuring various cellular components including RNAs, proteins, and metabolites (**Figure 2B**). There are successful examples of terpenoid production driven by systems biology to debottleneck synthesis pathway (Alonso-Gutierrez et al., 2015; Li et al., 2017; Wada et al., 2017). An integrated approach of multi-level Omics data has been pursued to obtain a desirable phenotype of host strain for production, because a genetic manipulation may have positive impact at one metabolic layer but a negative or neutral impact at another (Goh and Wong, 2016; Lechner et al., 2016). IPP toxicity always challenges the engineered microbial systems for terpenoid production. By multi-level Omics-integrated analysis, formation of isoprenyl-ATP analog is recognized as a cause of the IPP toxicity, which suggests potential engineering strategies for terpenoids production from *E. coli* (George et al., 2018).

## Consortia Process for Production of Terpenoids

Complicated structures of terpenoids are often a hurdle of the production using a single microbe. As *E. coli* is generally not a tractable host for P450 chemistry, it is difficult to produce terpenoids decorated by P450 from *E. coli*. Although an oxygenated taxanes is produced in *E. coli* with optimization of P450 expression and its reductase partner interaction (Biggs et al., 2016), it requires lots of elaborate endeavors and cannot be copied into an engineering of other P450s. It would be beneficial to engineer multiple organisms to carry out a complicated task together rather than an engineering of a single organism alone (Hays et al., 2015). With greater understanding of microbial traits, coordinated microbial consortia can be designed and built to address complex tasks. Expansion of metabolic capacities and improvement of production yields could be attained in the synthetic microbial communities along with simplification of engineering of a complicated long pathway to a few simple shorter pathways distributed in the community microbes. The synthetic microbial consortia can be classified an intraspecies homo-consortia and an interspecies hetero-consortia, of which each strain is assigned an individual role toward product synthesis (**Figure 2C**). A key issue of synthetic microbial consortia engineering is to balance each microbial population for a successful collaboration to attain the goal (Johns et al., 2016). In order to produce acetylated taxanes, the entire pathway was divided into taxadiene synthesis expressed in *E. coli* and P450 modification in *S. cerevisiae* (Zhou et al., 2015). *E. coli* was engineered to use xylose, but produce a wasteful acetate, while auxotrophic *S. cerevisiae* assimilated solely acetate for cell growth in presence of xylose as a sole carbon source. The mutualistic consortium could maintain a population balance of the co-cultured strains and doubled the production in comparison with a co-culture using glucose. Deeper understanding of autotrophy, interspecies cross-feed, and QS machinery would provide more exquisite approaches for production of complicated terpenoids using synthetic microbial consortia.

## Conclusion

Currently, many enabling technologies of synthetic biology allow us to tailor microbes for terpenoids production. Among the microbial species, *E. coli*, and yeasts have proved as the most attractive microbial platform, which can be conveniently developed to generate either bulk or value-added terpenoids owing to versatile enabling technologies for the microbes. A great potential of *E. coli* and yeasts as platform strains has been demonstrated with many successes of synthetic pathway rewiring, genome editing, and microbial consortia building for improvement of production. With deeper understanding of their metabolism, more promising approaches are expected to boost production of terpenoids in *E. coli* and yeasts.

## Author Contributions

CW and S-WK developed the ideas and drafted the manuscript. ML, J-BP, and S-HJ collected the literatures and drew the figures. GW, YW, and S-WK professionally edited the manuscript.

## Conflict of Interest Statement

The authors declare that the research was conducted in the absence of any commercial or financial relationships that could be construed as a potential conflict of interest.

## Footnotes

1 http://investors.amyris.com/press-releases
